# Effects of dicopper oxide and copper sulfate on growth performance and gut microbiota in broilers

**DOI:** 10.1016/j.psj.2021.101224

**Published:** 2021-04-27

**Authors:** A. Forouzandeh, L. Blavi, N. Abdelli, D. Melo-Duran, A. Vidal, M. Rodríguez, A.N.T.R. Monteiro, J.F. Pérez, L. Darwich, D. Solà-Oriol

**Affiliations:** ⁎ Animal Nutrition and Welfare Service (SNiBA), Departament de Ciència Animal i dels Aliments, Universitat Autònoma de Barcelona, 08193 Bellaterra, Spain; † Department of Animal Health and Anatomy, Universitat Autònoma de Barcelona, 08193 Bellaterra, Spain; ‡ PigCHAMP Pro Europa, Segovia, Spain; § Animine, Annecy, France

**Keywords:** copper, broiler, growth performance, ileal microbiota, antimicrobial resistance

## Abstract

An experiment was conducted to determine the effects of two sources of copper (**Cu**) from copper sulfate (**CuSO_4_**) and dicopper oxide (**Cu_2_O**, CoRouge) at three levels of inclusion (15, 75, and 150 mg/kg) on growth performance and gut microbiota of broilers. A total of 840 one-d-old male chickens (Ross 308) were weighed and randomly allocated to seven dietary treatments: negative control (**NC**, a basal diet without Cu addition), and the NC supplemented with 15, 75, or 150 mg Cu/kg from CuSO_4_ or Cu_2_O (12 replicate pens/treatment, 10 chicks per pen). Broilers were challenged by reusing an old litter with high concentrations in *Clostridium perfringens* to promote necrotic enteritis. Broiler performance was registered at d 21, 35, and 42. Excreta samples were collected at d 14, 28, and 42 for antimicrobial resistance (**AMR**) analyses. At d 43, one broiler per pen was euthanized to obtain ileal content for microbial characterization. Body weight d 35 and daily gain d 42 improved (*P* < 0.05) in Cu_2_O as Cu dose inclusion increased from 15 mg/kg to 150 mg/kg. Supplementation of 150 mg/kg of Cu from Cu_2_O decreased the abundance (*P* < 0.01) of some families such as Streptococcaceae and Corynebacteriaceae and increased the abundance (*P* < 0.05) of some commensal bacteria like Clostridiaceae and Peptostreptococcaceae. Phenotypic AMR was not different among treatments on d 14 and 28. Isolated *Enterococcus* spp. from broilers fed the NC diet on d 42 showed higher (*P* < 0.05) resistance to enrofloxacin, gentamicin, and chloramphenicol compared with Cu treatments. By contrast, the isolated *Escherichia coli* from broilers fed 150 mg/kg of Cu, either from CuSO_4_ or Cu_2_O, showed higher (*P* < 0.05) resistance to streptomycin and chloramphenicol compared to the NC. This study suggests that supplementing 150 mg/kg of Cu from Cu_2_O establishes changes in the gut microbiota by regulating the bacterial population in the ileum, which may explain the positive impact on broilers' growth performance.

## INTRODUCTION

Copper (**Cu**) is an essential trace mineral in the poultry diet (Davis and Mertz, 1987). It is involved in immune function and oxidation, plays a significant role in iron metabolism (Kim et al., 2008; Ognik et al., 2016), and allows optimal growth performance by maintaining body functions (Banks et al., 2004b). The copper requirement for broilers is 5 to 8 mg/kg diet according to NRC (1994) and 4 to 10 mg/kg according to FEDNA (2018), but the maximum dosage authorized by EFSA (2012) in the European Union is 25 mg/kg. However, in many non-EU countries, therapeutic doses (125−250 mg/kg of Cu) of Cu from copper sulfate pentahydrate are being widely used as a growth promoter and antibacterial feed additive (Pang and Applegate, 2006).

Therapeutic doses of Cu may improve growth performance in animals by modulating the microbial population within the gastrointestinal tract (Arias and Koutsos, 2006) and, therefore, improving nutrient absorption (Hawbaker et al., 1961; Bunch et al., 1965). On the other hand, high Cu dosages influence antibiotic resistance development (Poole, 2017), and pollute the environment through higher Cu excretion (Malan et al., 2015). However, the antibacterial properties of Cu may depend on its redox state: Cu(I), the reduced cuprous form, has a stronger antibacterial effect in anaerobic conditions than Cu(II), the oxidized cupric form (Dunning et al., 1998). Besides, the different solubility and bioavailability of Cu sources may affect intestinal microbiota in a different way (Pang et al., 2009).

Copper sulfate (**CuSO_4_**) is soluble in water (99%) and acidic solvents (Pang and Applegate, 2006) and has a Cu concentration of 25.4% (Baker, 1999). On the other side, dicopper oxide (Cu_2_O, CoRouge, Animine) is a water nonsoluble compound that has the highest Cu concentration in the market (75% of Cu). In a previous study, Hamdi et al., (2018) observed that therapeutic doses (150 mg/kg of Cu) of Cu_2_O in broilers diet increased their body weight (**BW**), however, when 150 mg/kg of Cu from CuSO_4_ was supplied growth performance was not modified, and feed efficiency reduced with 300 mg/kg addition. It was also suggested that excessive Cu accumulates in different organs, and free unbound copper in the blood may act as a strong oxidizing agent and cause a toxic response (Banks et al., 2004b; Reece et al., 2015). Nevertheless, there is no information about the effect of Cu_2_O on gut microbiota.

Taking into account all the effects, we have hypothesized that using the most effective source (Cu_2_O) could enhance performance at a therapeutic dose of 150 mg/kg, or even lower dosage, through changes in the gut microbiota. It was also hypothesized that differences between the sources may lead to a reduction in antimicrobial resistance (**AMR**) development caused by high Cu concentration.

Therefore, the objective of our study was to explore the effect of 75 or 150 mg/kg dose of Cu from Cu_2_O on growth performance, intestinal microbiota profile, and AMR when it is compared to CuSO_4_ in broilers challenged with recycled necrotic enteritis (**NE**) litter.

## MATERIALS AND METHODS

All experimental animal procedures were approved by the Animal Ethics Committee of the Universitat Autònoma de Barcelona and complied with the European Union guidelines for the care and use of animals in research (European Commision, 2010).

### Bird Management and Husbandry

The study was carried out at a commercial growing poultry unit (Tarragona, Spain). The room was provided with 84 solid-sided pens (0.8 × 1 m) in 4 lines of 21 pens divided by a central feeding aisle. A total of 840 one-d-old male chickens (Ross 308) were randomly allocated to one of 7 dietary treatments (12 replicate pens/treatment, 10 chicks per pen, and 0.64 m^2^ per chick) according to initial BW and continuously controlled over 42 d. The average temperature was maintained at 35 ± 1°C and was decreased gradually (at the rate of 3°C per wk) to 20°C until d 42. The light cycle was provided 24 h/d from d 1 to d 2, 23 h/d from d 3 to d 10, and 18 h/d from d 11 to the last day of the experiment. Broilers were challenged by a recycled NE litter.

### Necrotic Enteritis Challenge Procedure

The selection of the recycled litter material was made between four commercial poultry flocks based on signs of NE. The farm with the highest concentration of clinical NE and previously characterized for its content of mesophilic aerobic bacteria (> 10^5^ CFU/g), *Enterobacteriaceae* (5.2 × 10^3^ CFU/g), filamentous fungi and yeasts (2.2 × 10^3^ CFU/g), and *Clostridium perfringens* (5.6 × 10^4^ CFU/g) was selected. The floor area was covered with 10% clean wood shavings and 90% recycled litter material on the first day of the experiment. The challenging process comprised of exposing broilers to a contaminated litter characterized by high *Clostridium perfringens* counts was formerly used by Abdelli et al. (2020).

### Experimental Diets

A 3-phase feeding program was used, a starter phase from d 0 to d 21, a grower phase from d 22 to d 35, and a finisher phase from d 36 to d 42 (Table 1). Seven diets for each phase (21 diets in total) were prepared in a pelleted form (with a size of 1.8 mm for the starter phase and 3 mm for the grower and finisher). Dietary treatments were negative control (**NC**) diet without Cu supplementation and six additional diets in which 15, 75, or 150 mg/kg of Cu from CuSO_4_ (Copper sulfate, 24.1% Cu, Manica Cobre S.L, Spain) or Cu_2_O (CoRouge, 75.4% Cu, Animine, Sillingy, France) were added to the NC diet. The analyzed Cu concentration of each diet is presented in Table 2. The mineral-vitamin premix included in the diet was formulated and mixed without Cu. Diets were formulated to be isonutritive and to meet current estimates for nutrient requirements for growing broilers (FEDNA, 2018) and without antibiotics and growth promoters. Feed and water were offered *ad libitum*. Each diet was sampled in duplicate, grounded, and stored at 4°C for their subsequent analysis.

**Table 1:** Composition and nutrient content of the basal diet.

Item	Starter	Grower	Finisher
Ingredients, %			
Ground corn	59.99	60.68	61.01
Soybean meal 47%	33.40	31.22	29.62
Soybean oil	2.55	4.27	5.66
Monocalcium phosphate	1.37	1.29	1.20
Calcium carbonate	1.28	1.21	1.21
Mineral-vitamin premix^1^	0.30	0.30	0.30
Sodium chloride	0.31	0.30	0.24
_DL_-Methionine	0.25	0.25	0.24
_L_-Lysine.HCl	0.15	0.10	0.12
_L_-Threonine	0.05	0.05	0.06
Sodium bicarbonate	0.20	0.20	0.2
Choline chloride	0.15	0.13	0.14
Calculated composition, %			
ME Kcal/kg	2950	3050	3150
Crude Protein	21.20	20.00	18.50
Calcium	0.98	0.90	0.78
Phosphorus	0.68	0.65	0.62
Lysine	1.22	1.11	1.07
Methionine	0.56	0.54	0.52
Methionine + cysteine	0.91	0.87	0.84
Threonine	0.81	0.77	0.75
Tryptophan	0.23	0.23	0.21
Analyzed composition, %			
Dry matter	89.10	88.26	88.44
Crude Ash	5.69	5.48	5.08
Crude Protein	20.49	19.02	18.52
Crude Fat	5.43	6.73	7.76
Crude Fiber	3.04	2.96	3.43

Abbreviation: ME, metabolizable energy.

1 Provided per kg of diet: vitamin A (retinyl acetate), 17,000 IU; vitamin D_3_ (Cholecalciferol), 3,500 IU; vitamin E (dl-α-tocopheryl acetate), 15 IU; vitamin K_3_ (menadione sodium bisulfate), 2 mg; vitamin B_1_, 1.6 mg; vitamin B_2_, 4.16 mg; vitamin B_6_, 2 mg; vitamin B_12_, 0.012 mg; nicotinic acid, 21.2 mg; pantothenic acid (D-Ca pantothenate), 10.58 mg; biotin, 0.048 mg; folic acid, 0.8 mg; Zn (ZnO) 60.19 mg; Fe (FeSO_4_·7H₂O), 24 mg; Mn (MnSO₄·H₂O), 54.06 mg; I (KI), 0.6 mg; and Se (NaSeO_3_), 0.18 mg; antioxidant, 0.8 mg.

**Table 2:** Calculated and analyzed Cu concentration in the experimental diets.

	Cu level, mg/kg			
	Calculated	Analyzed^1^		
Item		Starter	Grower	Finisher
Negative Control	7	6	9	6
CuSO_4_	15	22	23	20
	75	78	103	88
	150	131	213	138
Cu_2_O	15	30	23	20
	75	95	84	81
	150	139	152	169

1 The values expressed as mean based on duplicate determinations.

### Performance Measurements and Sample Collection

All the birds were weighed individually on d 0, 21, 35, and 42, and feed intake was recorded at d 21, 35, and 42. The average daily gain (**ADG**), average daily feed intake (**ADFI**), and feed conversion ratio (**FCR**) were calculated at the end of each phase and for the global period. Mortality and cause of death were also recorded.

Excreta samples were collected at d 14, 28, and 42, from three animals per pen of all treatments (10 replicate pens/treatment), and a pool of excreta samples was made for each pen and day to analyze AMR. At d 43, 1 broiler per pen with a similar BW to the average of the pen was selected. The broilers were stunned using an electrical stunner (Reference: 105523, FAF, France) and immediately exsanguinated to obtain ileal content.

Subsequently, based on the performance results, ileal content and excreta samples of NC and 150 mg/kg of Cu from CuSO_4_ and Cu_2_O treatments were used for the analysis of microbiota 16S rRNA gene, and AMR, respectively.

### Chemical Analysis

All the diets were analyzed according to standard methods for dry matter (ISO, 1999), crude ash (ISO, 2002), and crude protein (ISO, 1997). Crude fat was analyzed with the Soxhlet method using Foss Soxtec/Hydrotec 8000 System for total fat analysis, consisting of Soxtec 8000 extraction unit and Hydrotec hydrolysis unit, (FOSS Analytical, Denmark). The crude fiber content was also measured using the Weende method (NF V03–040). The copper content in all the diets was determined using Inductively Coupled Plasma Optical Emission Spectroscopy (ICP-OES, model Optima 4300DV, PerkinElmer Inc.; Waltham, MA).

### Microbiota 16S rRNA Gene Analysis

#### Library Preparation and Sequencing

Bacterial DNA was taken out from 250 mg of ileal content following the manufacturer's instructions with the commercial MagMAX CORE Nucleic Acid Purification Kit 500RXN (Thermo Fisher, Barcelona, Spain). Mock community DNA was involved as a control (Zymobiomics Microbial Community DNA). Samples were amplified using specific primers to the V3-V4 regions of the 16S rRNA DNA (V3-V4-Forward 5′-TCGTCGGCAGCGTCAGATGTGTATAAGAGACAGCCTACGGGNGGCWGCAG-3′, V3-V4-Reverse 5′GTCTCGTGGGCTCGGAGATGTGTATAAGAGACAGGACTACHVGGGTATCTAATCC-3′) (Klindworth et al., 2013). The library preparation was performed in Microomics Systems SL (Barcelona, Spain).

### Amplicon Sequences Processing and Analysis

Forward and reverse reads of raw demultiplexed were processed by following the methods and pipelines as implemented in QIIME2 version 2019.4 with defaulting parameters unless indicated (Bolyen et al., 2019). DADA2 was used for quality filtering, denoising, pair-end merging, and amplicon sequence variant calling (**ASV**, i.e., phylotypes) using *qiime dada2 denoise-paired* method (Callahan et al., 2016). Q20 was used as a quality threshold to define read sizes for trimming before merging (parameters: –p-trunc-len-f and –p-trunc- len-r). Reads were truncated at the place when the 75th percentile Phred score felt below Q20 for both forward and reverse reads. After quality filtering steps, the average sample size of reads was resolved and phylotypes were detected. ASVs were aligned using the *qiime alignment mafft method* (Katoh and Standley, 2013). The alignment was used to generate a tree and to calculate phylogenetic relations between ASVs using qiime phylogeny FastTree method (Price et al., 2010). To even sample sizes for the diversity analysis using *qiime diversity core-metrics-phylogenetic* pipeline, ASV tables were subsampled without replacement. The sample with the smallest size was discarded to take advantage of the sequencing depth of the dataset. Afterward, subsampling to the next lowest sample size was used for each comparison. Unweighted and weighted Unifrac distances were calculated to compare community structure (Lozupone et al., 2011). Taxonomic assignment of ASVs was performed using a Bayesian Classifier trained with Silva V4 database (i.e., 99% OTUs database) using the *qiime feature-classifier classify-sklearn* method (Pedregosa et al., 2011). Unifrac distance matrices and ASV tables were used to calculate principal coordinates and construct ordination plots using the R software package version 3.6.0 (http://www.R-project.org).

### Antimicrobial Resistance Analysis

Excreta samples (10 replicate pens/treatment) were analyzed for microbiological isolation of *Enterococcus* spp. and *Escherichia coli* (***E. coli***), using Slanetz-Bartley (Oxoid, UK) for 48 h at 37 °C and McConkey agar plates (Oxoid, UK) for 24 hours at 37°C, respectively. Compatible colonies with *Enterococcus* spp. and *E. coli* were confirmed and identified by PCR (Dutka-Malen et al., 1995). Genotypic AMR analysis was done in all the bacterial isolates to detect the resistance genes for vancomycin (vanC1 and vanC2) (Dutka-Malen et al., 1995; Kariyama et al., 2000), tetracycline tet(**M**), and erythromycin erm(**B**) (Jacob et al., 2008). The detection of extended-spectrum beta-lactamases (**ESBL**) [blaSHV, blaCTX-M, blaCMY1, blaCMY2, and blaTEM] and carbapenemase-resistance (**OXA-48**) genes was performed as previously described by Vidal et al. (2020). Also, copper (**tcrB**) and zinc (**czcA**) resistance genes were analyzed, as previously described by Hasman et al. (2006).

In parallel, all *Enterococcus* spp. and *E. coli* isolates were tested for phenotypic antimicrobial sensitivity using the disk diffusion method, described by Bauer et al., (1966). Thirteen antimicrobial agents were used: penicillin G (10µg, Oxoid, Basingstoke, UK) ampicillin (25 µg, BD), imipenem (10 µg, BD), vancomycin (30 µg, BD), erythromycin (15 µg, BD), tetracycline (30 µg, BD), ciprofloxacin (5 µg, BD), enrofloxacin (5 µg, BD), clindamycin (2 µg, BD), gentamicin (10 µg, BD), kanamycin (30 µg, BD), streptomycin (10 µg, BD) and chloramphenicol (30 µg, BD). Cut-off values were those defined by the Clinical Laboratory and Standards Institute.

Also, minimum inhibitory concentration tests were performed to assess the susceptibility of *Enterococcus* spp. and *E. coli* strains to copper (II) sulfate pentahydrate (**CuSO_4_·5H_2_O**) using the broth microdilution method as previously reported (Hasman et al., 2006).

### Statistical Analysis

Growth performance data were analyzed as a complete randomized design with ANOVA using the GLM procedure of SAS software (SAS 9.4 Institute Inc., Cary, NC). Homoscedasticity and variances normal distribution were checked before the analysis using the Shapiro-Wilk test and Levene's test from UNIVARIATE and GLM procedures, respectively. For growth performance parameters, the model included Cu source, Cu dose, and their interaction as a main effect and period as a random effect. The LSMeans statement was used to calculate mean values for each parameter. The AMR data were analyzed using the chi-squared test (Fisher Exact Test). For microbiota, Alpha and Beta diversity were analyzed using Vegan package and taxa differences with the MetagenomeSeq package in open source software RStudio v.3.5.1. Alpha diversity was calculated with raw counts based on Simpson, Shannon, and Inverse-Simpson estimators. Beta diversity was evaluated by multivariate ANOVA based on dissimilarities through envfit and adonis function. Finally, differential abundance analysis was performed with taxa relative abundances under a zero-inflated log-normal mixture model, *P*-values were corrected by the false-discovery rate with metagenomeseq package (Paulson et al., 2017).

The experimental unit was the replicate, and statistical significance and tendencies were considered at *P* ≤ 0.05 and 0.05 < *P* ≤ 0.10, respectively.

## RESULTS

### Growth Performance

Growth performance was lower than Ross 308 standards, which confirmed that the experimental challenge impaired the growth of the animals (Table 3). The mortality rate was 2.5% for the overall experiment, with no differences among the dietary treatments (results not presented). Broilers fed 150 mg/kg of Cu from Cu_2_O had higher (*P* = 0.033) BW at d 35, and tended to have higher BW (*P* = 0.053) at d 42 than broilers fed 15 mg/kg of Cu from Cu_2_O, a result which was not observed with CuSO_4_. Broilers fed 150 mg/kg of Cu from Cu_2_O had higher ADG (*P* = 0.019) than birds fed 15 mg/kg of Cu_2_O, or NC at d 42.

**Table 3:** Growth performance (BW, ADFI, ADG, and FCR) of broilers fed dietary treatments.^1^

		NC	CuSO_4_, mg/kg			Cu_2_O, mg/kg			SEM	*P*-value		
Item			15	75	150	15	75	150		Source	Dose	Source*Dose
BW, g	d 21	628.7	639.1	629.6	630.9	601.5	631.0	649.7	11.92	0.608	0.421	0.121
	d 35	1684.5^ab^	1718.3^ab^	1715.4^ab^	1710.7^ab^	1616.0^b^	1718.2^ab^	1783.6^a^	29.21	0.748	0.038	0.033
	d 42	2420.8^xy^	2505.6^xy^	2489.7^xy^	2473.8^xy^	2404.4^y^	2514.9^xy^	2585.5^x^	37.51	0.741	0.025	0.053
ADG, g/d	d 0-21	28.1	28.4	28.1	28.2	26.8	28.1	29.1	0.56	0.647	0.319	0.166
	d 21-35	75.0	77.0	77.6	76.7	72.5	77.7	80.6	1.70	0.912	0.060	0.113
	d 35-42	105.2	112.5	108.5	112	108.2	113.8	114.6	2.57	0.615	0.019	0.307
	d 0-42	56.6^b^	58.6^ab^	58.3^ab^	57.9^ab^	55.6^b^	58.9^ab^	60.5^a^	0.88	0.932	0.013	0.019
												
ADFI, g/d	d 0-21	43.3^AB^	44.0^A^	43.0^AB^	41.6^B^	41.2^B^	41.8^AB^	43.3^AB^	0.53	0.127	0.371	0.001
	d 21-35	133.1^ab^	141.3^a^	133.2^ab^	137.2^ab^	131.1^b^	131.5^b^	136.7^ab^	2.03	0.032	0.062	0.043
	d 35-42	180.6	194.2	187.4	187.6	183.3	190.8	189	3.49	0.545	0.052	0.179
	d 0-42	96.1^B^	101.5^A^	97.4^AB^	97.1^AB^	94.8^B^	96.5^B^	98.7^AB^	1.16	0.076	0.267	0.004
												
FCR, g/g	d 0-21	1.54	1.55	1.53	1.48	1.54	1.49	1.49	0.02	0.425	0.035	0.612
	d 21-35	1.78	1.84	1.73	1.80	1.81	1.70	1.70	0.04	0.142	0.027	0.594
	d 35-42	1.72	1.73	1.73	1.68	1.70	1.69	1.65	0.04	0.374	0.584	0.960
	d 0-42	1.70	1.73	1.67	1.68	1.71	1.64	1.63	0.02	0.063	0.004	0.663

Abbreviations: ADG, average daily gain; ADFI, average daily feed intake; BW, body weight; FCR, feed conversion ratio; NC, negative control.

^a-b^Means with different superscripts within a row indicate a significant difference of source*dose (*P* ≤ 0.05).

^x-y^Means with different superscripts within a row indicate a tendency toward the significance of source*dose (*P* ≤ 0.1).

1 Data are means of 12 replicates per treatment.

Supplementation of Cu from Cu_2_O, irrespective of dose, tended to have lower ADFI (*P* = 0.076) and FCR (*P* = 0.063) than CuSO_4_ supplementation at d 42.

### Microbiota 16S rRNA Gene Analysis

Both alpha (Shannon, Simpson, and Inverse Simpson index) and beta diversity metrics were used to estimate microbial communities' diversity. Alpha diversity indices showed higher diversity and evenness (*P* < 0.05) in ileal microbiota of chickens fed 150 mg/kg of Cu from Cu_2_O compared with NC and 150 mg/kg of Cu from CuSO_4_ in Shannon index and the NC diet with all the indexes (Table 4). However, there were no differences in beta diversity among treatments (*P_ENVFIT_ =* 0.4, data not shown).

**Table 4:** Differences in α-diversity indices in ileal microbiota of broilers fed the NC diet or 150 mg/kg of Cu from CuSO_4_ and Cu_2_O at d 42.^1^

	NC	CuSO_4_, mg/kg	Cu_2_O, mg/kg	SEM	*P*-value
Item		150	150		
Shannon	1.41^B^	1.62^B^	2.13^A^	0.13	0.003
Simpson	0.57^B^	0.65^AB^	0.81^A^	0.05	0.007
Invsimpson	3.01^b^	3.30^ab^	4.99^a^	0.54	0.030

Abbreviation: NC, negative control.

^a-b^ Means with different superscripts within a row indicate significant differences (*P* ≤ 0.05).

1 Data are means of 12 replicates per treatment.

**Figure 1:**
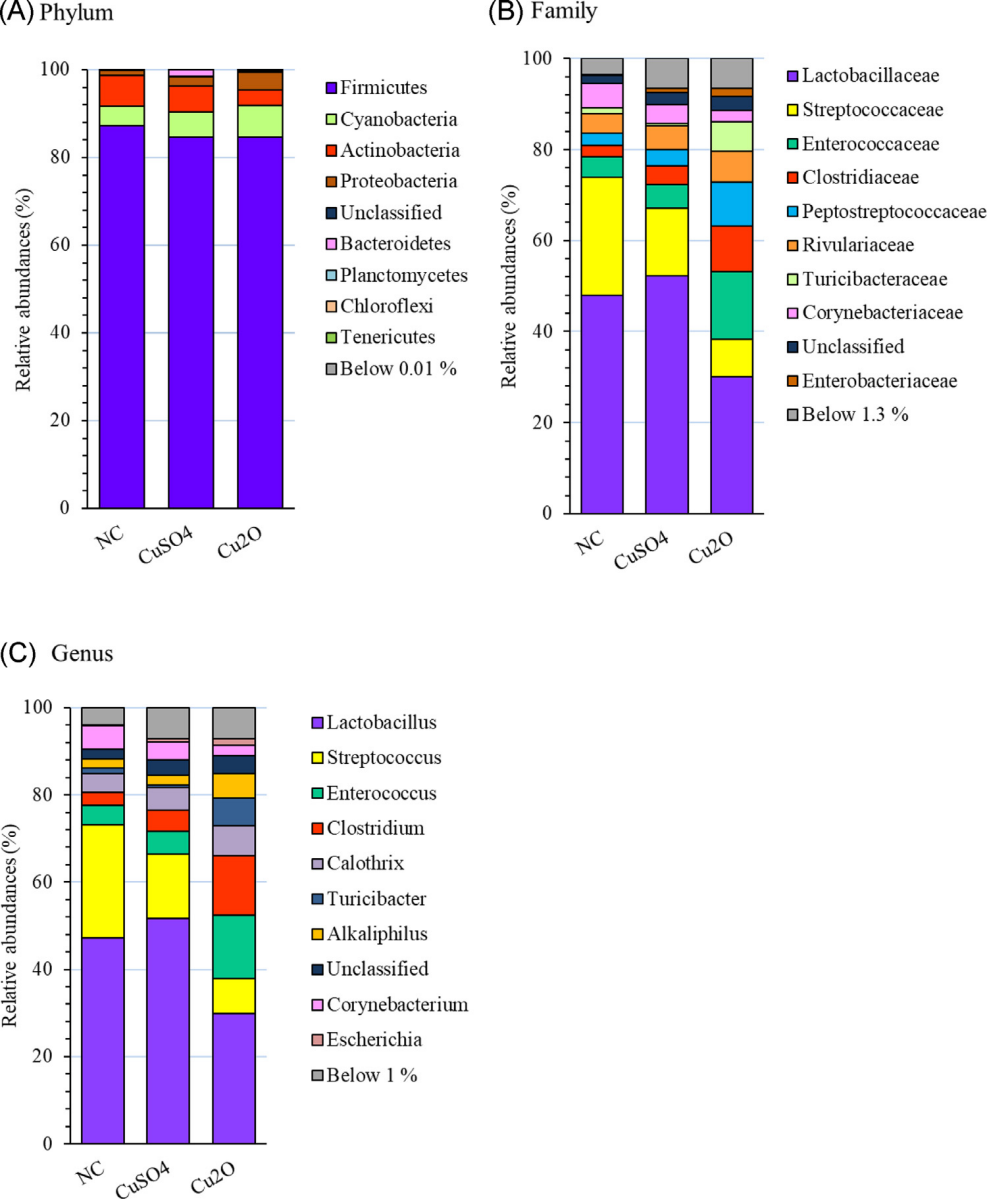
Relative abundance (%) of Top 10: phyla (A); families (B); and genera (C), in the ileum of different experimental groups. The rest of the taxonomic groups are pooled together (those representing less than a mean of 0.01, 1.3, and 1% of phyla, families, and genera, respectively). Abbreviations: NC, negative control; CuSO_4_ = 150 mg/kg of Cu from CuSO_4_; Cu_2_O, 150 mg/kg of Cu from Cu_2_O.

The relative abundance of phyla, families, and genera detected among the experimental groups are illustrated in Figure 1. Among 25 recognized phyla, Firmicutes was the major phyla (average 85.47%), followed by Cyanobacteria and Actinobacteria (average 5.81% and 5.49%, respectively). At the family level, out of 222 different families, 92% of the operational taxonomic unit (**OTU**) was allocated to 9 families of Lactobacillaceae (30−53%), Streptococcaceae (8−26%), Enterococcaceae (4−15%), Clostridiaceae (2−10%), Peptostreptococcaceae (3−10%), Rivulariaceae (4−7%), Turicibacteraceae (1−6%)*,* Corynebacteriaceae (2−5%), and Enterobacteriaceae (0.2−1.7%), respectively. At the genus level, 85.88% of the OTU was assigned to 7 genera of *Lactobacillus, Streptococcus, Enterococcus, Clostridium, Calothrix, Turicibacter*, and *Alkaliphilus*.

A more in-depth examination of the individual metagenomics profile changes was detected on the dietary treatments using log_2_ changes.

**Figure 2:**
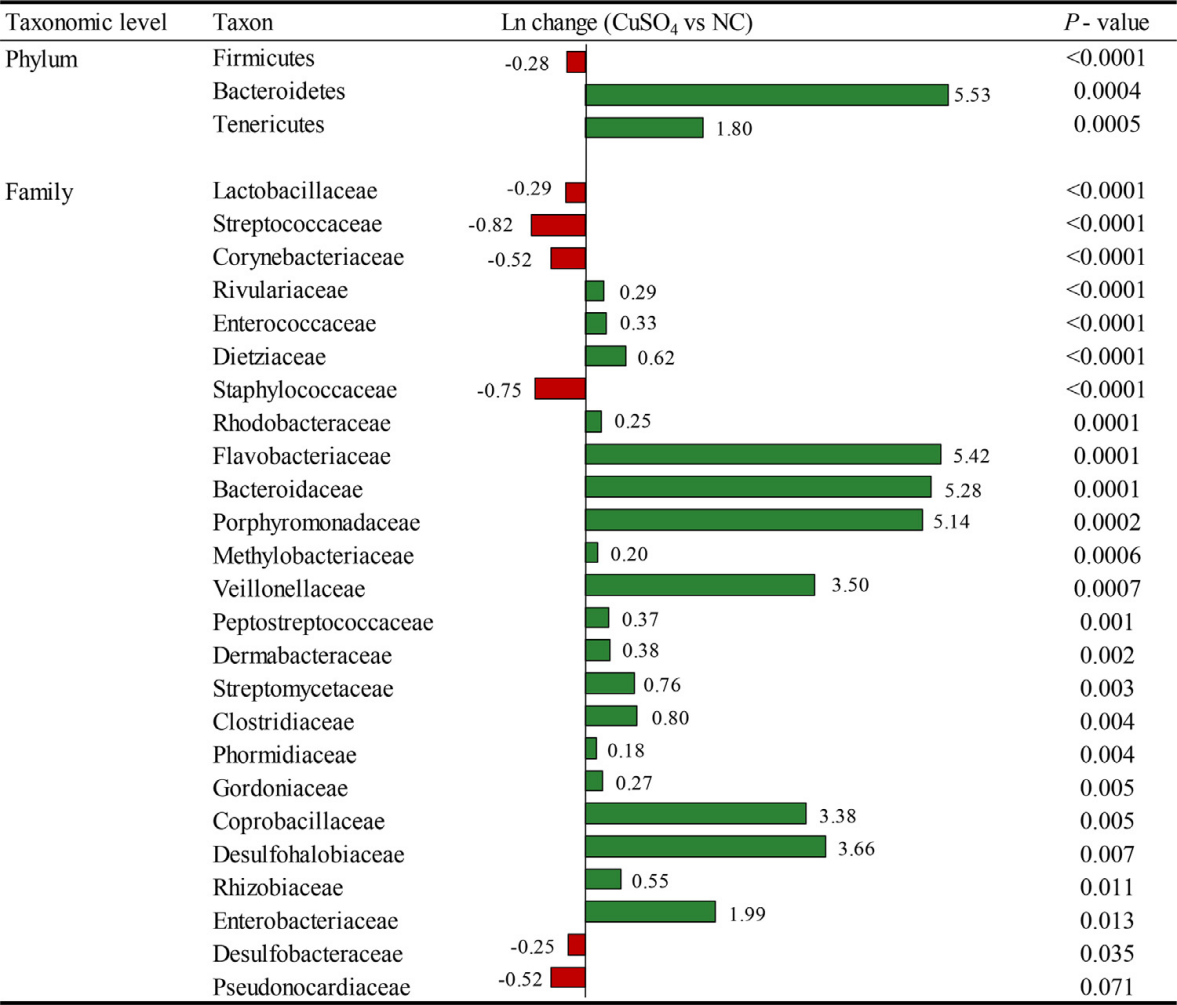
Differentially abundant taxa at the phylum and family level from the ileum on d 42 between 150 mg/kg of Cu from CuSO_4_ and NC. Positive values (green color) and negative values (red color) indicate higher and lower abundance, respectively. Taxa are sorted by level of significance (from higher to lower). Data are means of 12 observations per treatment. Abbreviation: NC, negative control.

Broilers fed 150 mg/kg of Cu from CuSO_4_ levels compared with those fed the NC (Figure 2) had significant differences in the relative abundance of Firmicutes (0.28 fold decrease; *P* < 0.0001) phyla, and some main families like Lactobacillaceae (0.29 fold decrease; *P* < 0.0001), Streptococcaceae (0.82 fold decrease; *P* < 0.0001), Corynebacteriaceae (0.52 fold decrease; *P* < 0.0001), Enterococcaceae (0.33 fold increase; *P* < 0.0001), Peptostreptococcaceae (0.37 fold increase; *P* = 0.001), Clostridiaceae (0.80 fold increase; *P* = 0.004), and Enterobacteriaceae (1.99 fold increase; *P* = 0.013).

**Figure 3:**
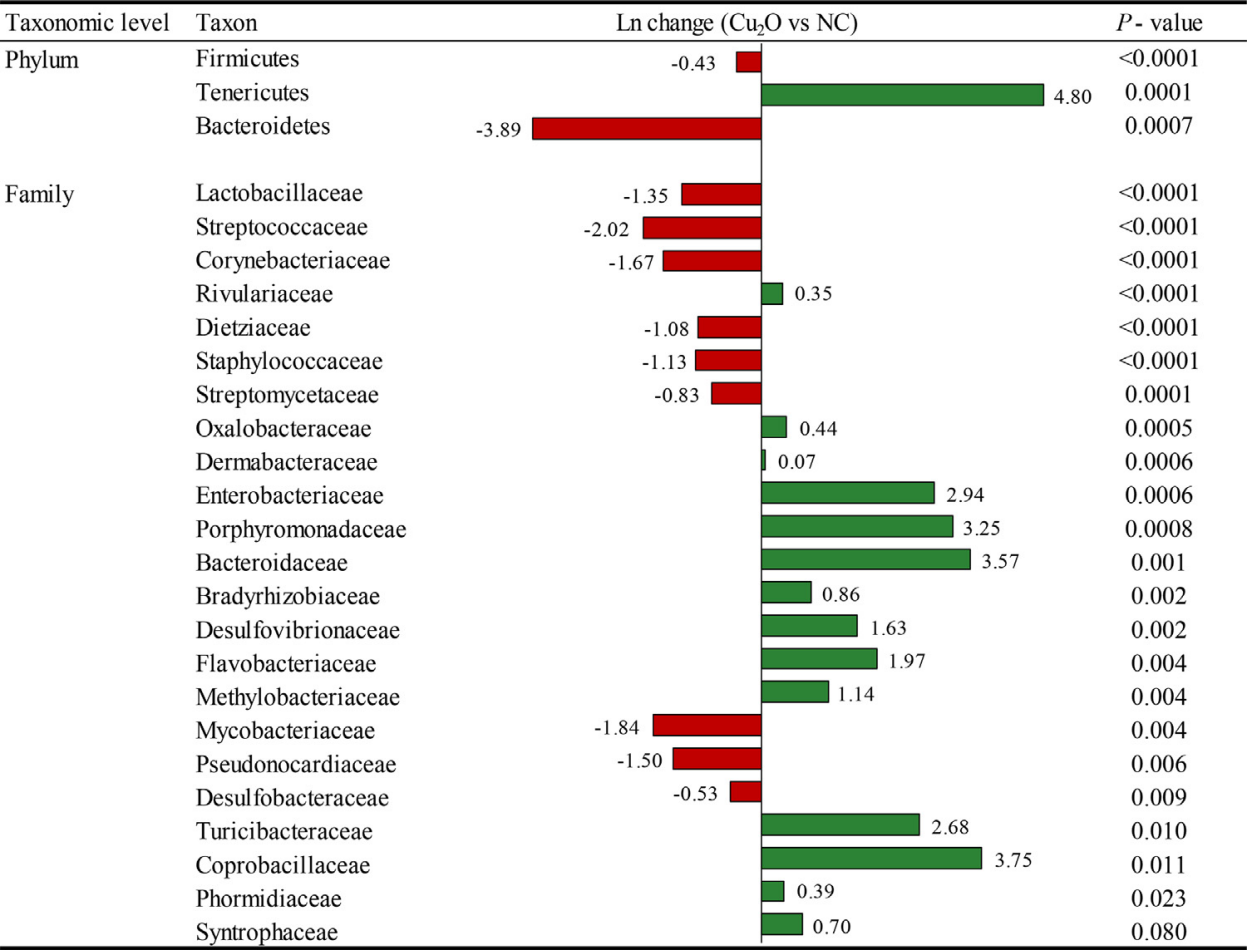
Differentially abundant taxa at the phylum and family level from the ileum on d 42 between 150 mg/kg of Cu from Cu_2_O and NC. Positive values (green color) and negative values (red color) indicate higher and lower abundance, respectively. Taxa are sorted by level of significance (from higher to lower). Data are means of 12 observations per treatment. Abbreviation: NC, negative control.

Broilers supplementation with 150 mg/kg of Cu from Cu_2_O significantly changed the abundance of Firmicutes (0.43 fold decrease; *P* < 0.0001) phyla, and families of Lactobacillaceae (1.35 fold decrease; *P* < 0.0001), Streptococcaceae (2.02 fold decrease; *P* < 0.0001), Corynebacteriaceae (1.67 fold decrease; *P* < 0.0001), and Enterobacteriaceae (2.94 fold increase; *P* = 0.0006), compared with broilers fed the NC diet (Figure 3).

**Figure 4:**
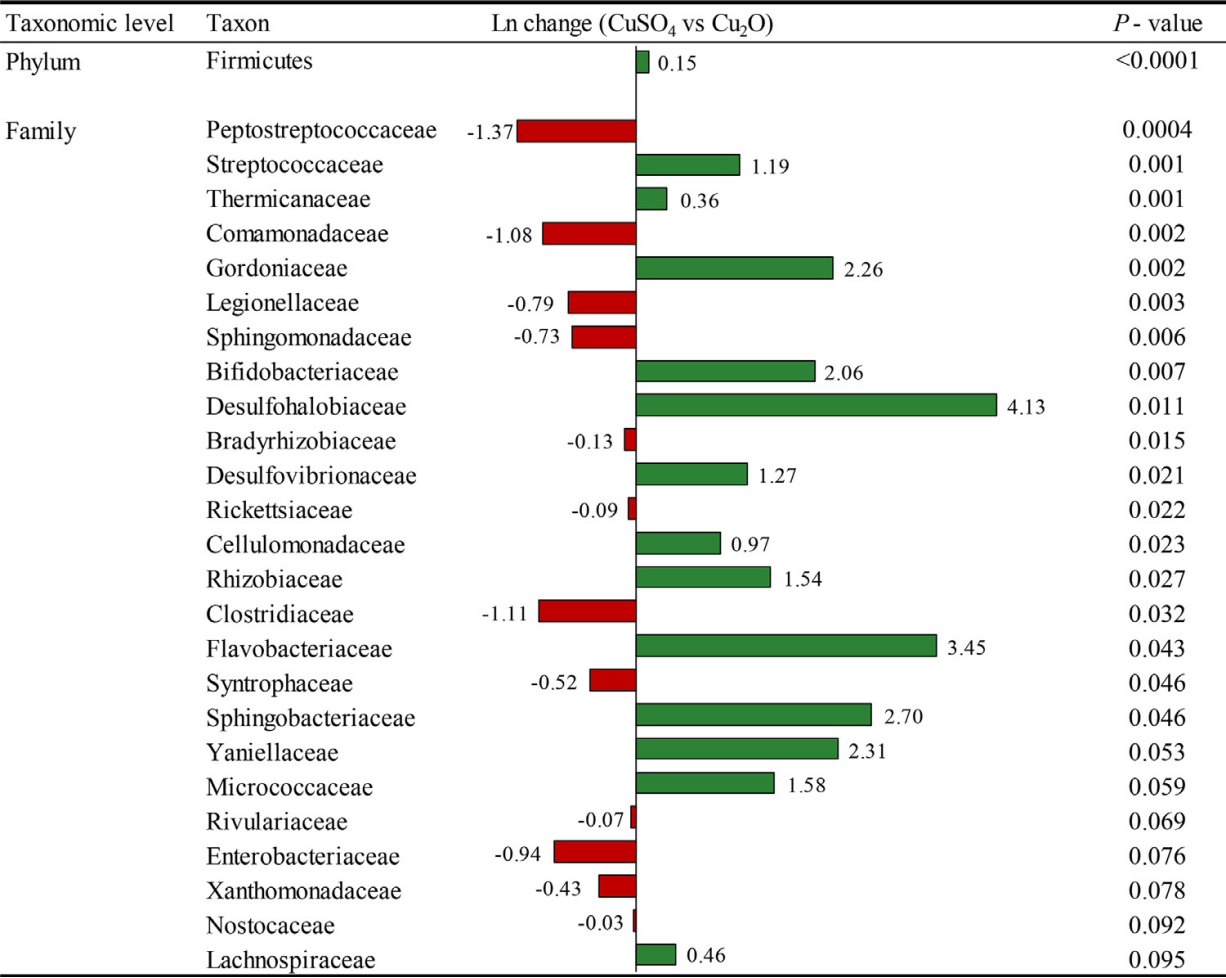
Differentially abundant taxa at the phylum and family level from the ileum on d 42 between 150 mg/kg of Cu from CuSO_4_ and Cu_2_O. Positive values (green color) and negative values (red color) indicate higher and lower abundance, respectively. Taxa are sorted by level of significance (from higher to lower). Data are means of 12 observations per treatment.

The comparison between Cu sources revealed that the addition of Cu at 150 mg/kg from Cu_2_O increased the abundance of Peptostreptococcaceae (1.37 fold; *P* = 0.0004), Clostridiaceae (1.11 fold; *P* = 0.032), and tended to increased the abundance of Enterobacteriaceae (0.94 fold; *P* = 0.076), but reduced the amount of Firmicutes (0.15 fold; *P* < 0.0001) phyla, and Streptococcaceae (1.19 fold; *P* = 0.001) family compared with 150 mg/kg of Cu from CuSO_4_ (Figure 4).

### Antimicrobial Resistance Analysis

*E. coli* was isolated from more than 80% of the excreta samples and *Enterococcus* spp. from all samples. As regards to *Enterococcus* spp., *E. faecalis* was the most frequently isolated, representing 70% (in the CuSO_4_ group) and 90% (in both NC and Cu_2_O groups) of the total isolates at d 42. Interestingly, *E. faecalis* detection was increasing according to days of the study (almost exclusively isolated from samples at d 42, and only detected in 2 samples from the CuSO_4_ at d 14 and 28).

In the genotypical analysis, isolates were negative for ESBL and OXA-48 genes. The percentage of resistant strains of *Enterococcus* spp. was higher for the rest of the studied genes, in all the treatments, and days compared to *E. coli* strains (Table 5). Moreover, the frequency of tcrB resistant strains had an increasing trend over time in all treatments (*P* > 0.1). For the *E. coli* isolates, the rate of vancomycin-resistant strains was lower (< 20%) for vanC1 and vanC2 genes in all treatments and days. VanA and VanB genes were not detected in any isolate.

**Table 5:** Genotypical antimicrobial resistance in isolates of *Enterococcus* spp. and isolates of *E. coli* of broilers fed the NC diet and 150 mg/kg of Cu from CuSO_4_ and Cu_2_O at d 14, 28, and 42.^1^

	Day 14			Day 28			Day 42		
AMR genes^2^	NC	CuSO_4,_ mg/kg	Cu_2_O, mg/kg	NC	CuSO_4_, mg/kg	Cu_2_O, mg/kg	NC	CuSO_4_, mg/kg	Cu_2_O, mg/kg
*Enterococcus* spp.									
vanC1	70%	70%	100%	30%	20%	10%	100%	100%	70%
vanC2	60%	70%	100%	90%	90%	80%	100%	100%	100%
tetM	100%	90%	100%	100%	100%	100%	100%	100%	100%
ermB	100%	100%	100%	100%	100%	100%	100%	100%	100%
ESBL	0%	0%	0%	0%	0%	0%	0%	0%	0%
OXA-48	0%	0%	0%	0%	0%	0%	0%	0%	0%
tcrB	90%	80%	80%	80%	60%	90%	100%	100%	100%
czcA	10%	10%	0%	0%	10%	0%	50%	40%	0%
*E. coli*									
vanC1	20%	0%	0%	0%	20%	0%	0%	0%	0%
vanC2	20%	0%	30%	0%	10%	0%	0%	0%	0%
tetM	89%	10%	0%	89%	90%	86%	0%	0%	0%
ermB	78%	100%	10%	78%	80%	100%	50%	70%	20%
ESBL	0%	0%	0%	0%	0%	0%	0%	0%	0%
OXA-48	0%	0%	0%	0%	0%	0%	0%	0%	0%
tcrB	0%	0%	0%	0%	10%	0%	70%	20%	40%
czcA	100%	50%	50%	78%	100%	43%	0%	0%	0%

1 Data are means of 10 replicates per treatment.

2 Antimicrobial resistance genes: vancomycin (vanC1, and vanC2); tetracycline (tetM); erythromycin (ermB); penicillin, aminopenicillin and last generation cephalosporine (ESBL); imipenem (OXA-48); Cu (tcrB); Zinc (czcA).

**Figure 5:**
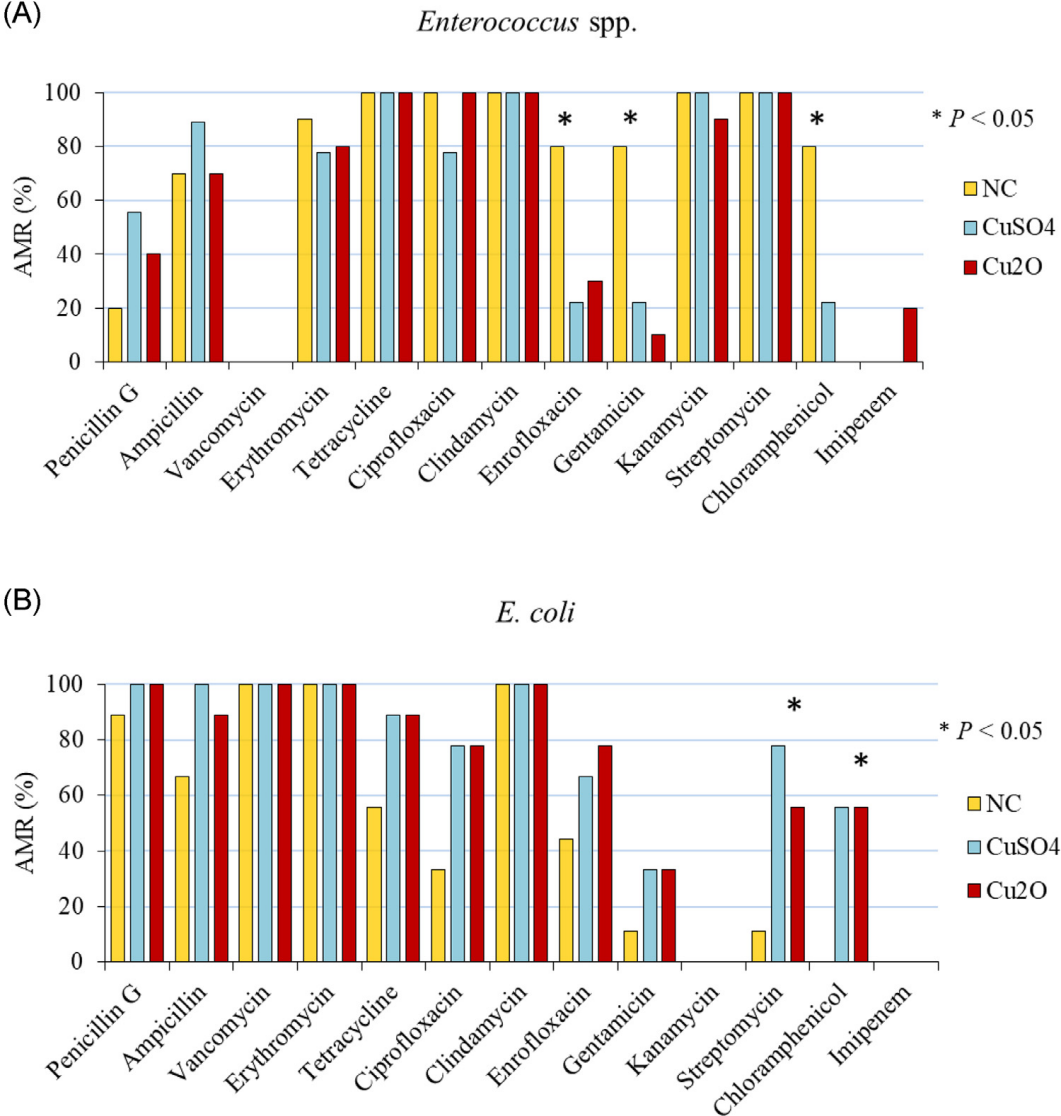
Phenotypic antimicrobial resistance in *Enterococcus* spp. isolates (A), and E. coli isolates (B) of broilers fed the NC diet and 150 mg/kg of Cu from CuSO_4_ and Cu_2_O at d 42. Abbreviations: NC, negative control; AMR, antimicrobial resistance.

There were no differences among dietary treatments on the phenotypic AMR at d 14 and 28. However, for the isolated *Enterococcus* spp., broilers fed the NC diet had higher (*P* < 0.05) resistance to enrofloxacin, gentamicin, and chloramphenicol compared to animals fed 150 mg/kg of Cu from CuSO_4_ and Cu_2_O on d 42 (Figure 5A). Conversely, the addition of 150 mg/kg of Cu from CuSO_4_ and Cu_2_O in the diet increased the *E. coli* resistance to streptomycin (78 and 56%, respectively) and chloramphenicol (56% on average) compared to the NC diet (11 and 0%, respectively; Figure 5B). Remarkably high levels of AMR were observed in all *E. coli* strains, even in the NC group.

## DISCUSSION

### Copper Effects on Growth Performance

In this experiment, *Clostridium perfringens* challenge established by reusing 90% recycled commercial litter resulted in reduced growth performance in comparison with the standard Ross 308 values (17.8% decrease); this result is in line with Abdelli et al. (2020) who observed a reduction of 21% by reusing commercial litter with NE. In this frame, our result revealed that Cu supplementation with Cu_2_O at 150 mg/kg dose increased the BW up to 10% at d 35 and numerically improved BW up to 7.5% at d 42 compared with 15 mg/kg dose. Whereas, supplied Cu as CuSO_4_ has not modified growth performance at therapeutic doses (150 mg/kg). A numerical improvement of ADFI (up to 2%) and FCR (up to 1.8%) was also observed with Cu_2_O supplementation in comparison to CuSO_4_.

The hypotheses by which Cu stimulates growth include regulation of intestinal microflora (Pang et al., 2009), enhancement of neuropeptide Y and its mRNA expression level (Li et al., 2008), and improvement of dietary fat digestibility as a result of stimulated lipase and phospholipase activities (Luo and Dove, 1996).

Previous researches have described that dietary Cu can be beneficial for growth performance when fed over the minimum requirements in poultry and swine. In a study by Arias and Koutsos (2006) supplementing broilers' diet with 188 mg/kg Cu from CuSO_4_ or tribasic copper chloride was also improved growth compared with those fed a non-supplemented diet, and growth improvement was the same with supplementation of sub-therapeutic antibiotics at d 45 under immune-challenging conditions (recycled vs. fresh litter). An 8.9% growth improvement and decreased FCR was observed by Samanta et al. (2011) when broilers fed 150 mg/kg of CuSO_4_ for 42 days. Similar positive effects of Cu on pigs were reported by Villagómez-Estrada et al. (2020), where 160 mg/kg Cu from CuSO_4_ or Cu hydroxychloride was able to increase performance at d 42.

The Cu response may, however, depend on the source. In an experiment conducted by Hamdi et al. (2018), dietary level of 150 mg/kg of Cu from Cu_2_O increased BW at d 35, whereas supplied Cu as CuSO_4_ at the same dosage did not improve growth performance compared with 15 mg/kg. Our findings were in line with Hamdi et al. (2018). In another study on broilers, Lu et al. (2010) indicated that adding 200 mg/kg of Cu from tribasic copper chloride improved ADG without increasing ADFI compared with 200 mg/kg of Cu from CuSO_4_ or other doses of both sources.

The negligible impact of CuSO_4_ on growth compared with other sources of Cu can be attributed to 1) damage to the mucosa and muscular layer in the intestinal tract (Chiou et al., 1999); 2) higher solubility (Pang and Applegate, 2006); 3) higher oxidation (Miles et al., 1998); 4) reduced phytase efficacy and decreased apparent phosphorus retention; and 5) toxicity (Banks et al., 2004a; Lu et al., 2010; Hamdi et al., 2018).

### Copper Effects on the Gut Microbiota Profile

One of the growth-promoting actions of Cu has been credited to its antimicrobial effect in the gastrointestinal tract. Copper ions are toxic and can effectively kill bacteria or mold by denaturation or an oxidation mechanism (Kim et al., 2007; Lok et al., 2007). The electrostatic attraction combines ionic Cu with the plasma membrane and results in the cell membrane penetration through opening or closing of the membrane channel. This process leads to the leakage of intracellular ions and low molecular-weight metabolites by altering the permeability of cellular membranes (Tong et al., 2005). Meanwhile, Cu^2+^ enters into the cell, induces plasmid DNA degradation (Giannousi et al., 2014), and leads to bacterial death (Tong et al., 2005).

Some researchers have reported that high dietary Cu has affected gut microbiota profile and reduced the growth of pathogenic bacteria in animals (Højberg et al., 2005; Zhang et al., 2017). Xia et al. (2004) reported that the positive effect of Cu on weight gain in broiler chickens might be an outcome of the significant reduction of the total pathogenic organism in the gut that intervenes with weight gain.

The analysis of ileal microbiota in the present study showed significant changes in some families of the gastrointestinal tract in broilers fed Cu. Supplementation of Cu (CuSO_4_ or Cu_2_O) in broilers' diet compared with the non-supplemented diet suppressed the abundance of Streptococcaceae*.* The genera *Streptococcus* is active in the process of simple sugar fermentation into lactate (Garvie, 1980; Zoetendal et al., 2012). Whereas, some species of the genus (e.g., *Streptococcus bovis*) are considered as major opportunistic pathogens, which can result in many diseases (Abdulamir et al., 2011; Munita et al., 2012; Qiao et al., 2014). A decreased *Streptococcus* abundance in colonic microbiota and increased growth performance have been observed in pigs fed 160 mg/kg of Cu (Villagómez-Estrada et al., 2020). Copper supplementation also declined the abundance of Corynebacteriaceae. Members of this family have been positively correlated with a wide range of severe infections, including opportunistic infections in both humans and animals (Prada et al., 1994; Zhi et al., 2017). Therefore, a lower proportion of Corynebacteriaceae may indicate a healthier intestinal environment.

Another family that responded to the treatments was Enterobacteriaceae, whose abundance was increased by Cu supplementation. The family Enterobacteriaceae includes 51 genera, which consist of commensal and pathogenic microorganisms (Janda, 2006). Opportunistic pathogens, for example, *Citrobacter, Enterobacter, Escherichia, Klebsiella, Serratia,* and *Proteus* have been associated with diarrhea, urinary tract infections, mastitis, arthritis, and meningitis (Fairbrother et al., 2005; Nagy and Fekete, 2005). However, low levels (0.86 to 1.52%) of the mentioned pathogens were present in broilers fed Cu supplementation.

The reduction of families containing pathogenic bacteria as a result of high Cu supplementation may lay the ground for the growth of other families. Adding 150 mg/kg of Cu from CuSO_4_ into the diet promoted the colonization of Enterococcaceae, Peptostreptococcaceae, and Clostridiaceae compared with the non-supplemented diet.

Enterococcaceae (*Enterococcus* spp.*)* belongs to the group of lactic acid bacteria. The genera consist of more than 20 species (Gomes et al., 2008). Some strains of this genus are capable of inhibiting the development of specific pathogens (Foulquié Moreno et al., 2006), and exhibit probiotic properties (Ó Cuív et al., 2013; Yadav and Jha, 2019). Peptostreptococcaceae and Clostridiaceae are members of Firmicutes phylum. Peptostreptococcaceae reported as normal commensal bacteria with a higher proportion in the gut microbiota of healthy animals than those experiencing dysbiosis of the intestinal microbiota. It indicates that this family helps preserve gut homeostasis (Fan et al., 2017). Clostridiaceae is one of the potential phylotypes involved in butyrate production from glucose, succinate, and lactate (Esquivel-Elizondo et al., 2017). Also, it has been highly correlated to protein and fat digestibility in dogs (Bermingham et al., 2017). The abundance of Peptostreptococcaceae and Clostridiaceae families have been previously shown to be associated with improved performance in broilers fed a mixture of organic acids with aromatic compounds or organic acids with medium-chain fatty acid plus aromatic compound (Abdelli et al., 2020).

On the other hand, the abundance of Lactobacillaceae was remarkably greater in broilers without Cu supplementation than those supplemented with Cu (CuSO_4_ or Cu_2_O). Lactobacillaceae *(****Lactobacillus spp.****)* is one of the main lactic acid-producing bacteria, and the primary end product of these bacteria is lactic acid (Garvie, 1980; Rajilić-Stojanović and de Vos, 2014) which has positive effects on growth. Interestingly, in a study by Gharib-Naseri et al. (2019), it has been asserted that increased *Lactobacillus* in the intestine may not indicate a healthier gut. Modified microbiota composition in challenging conditions could affect available nutrients for bacteria, and therefore, bacterial dynamics in the intestine (Stanley et al., 2012). Similar results were published by Park and Kim (2018). The authors reported an increase in the *Lactobacillus* population in the ileal digesta of broilers that received essential oils, but their BW were not significantly different from broilers in the non-supplemented group.

Comparing ileal microbiota of broilers fed Cu sources indicated that supplementation of Cu from Cu_2_O was more effective than CuSO_4_ towards the reduction of Streptococcaceae and development of Peptostreptococcaceae and Clostridiaceae which have beneficial properties. Bactericidal action of Cu contributed to the concentration of free ionic Cu in solution (Menkissoglu and Lindow, 1991). Therefore, reduced copper states, such as Cu_2_O, can provide Cu ion release more sustainably (Ren et al., 2011), and may exhibit a higher antibacterial activity (Dunning et al., 1998). Moreover, microbiota from the Cu_2_O treatment group was more diverse, and OTU's were more evenly distributed, compared to the NC and CuSO_4_ treatment groups. A correlation between FCR and richness and evenness indices has been previously observed by Stanley et al. (2016). In their experiments, broilers with low FCR showed higher diversity than those with high FCR. Furthermore, in a recent study, Villagómez-Estrada et al. (2020) discuss that reduction of opportunistic pathogens from one hand and development of saprophytic bacteria from the other hand, could lead to a significant improvement in intestinal nutrient absorption and, eventually, feed efficiency in pig fed 160 mg/kg of Cu from CuSO_4_ or Cu hydroxychloride.

In agreement with Villagómez-Estrada et al., (2020), our results suggest that adding 150 mg/kg of Cu, particularly Cu_2_O, appears to improve intestinal microbiota profile and enhance chickens' performance by increasing the abundance of reportedly beneficial bacteria, such as Peptostreptococcaceae and Clostridiaceae, reducing the colonization of harmful bacteria, and increasing the diversity and evenness of ileal microbiota. However, further work is s required to understand how these changes in bacterial composition relate to metabolic changes in the host that ultimately lead to improved performance.

### Copper Effects on the Antimicrobial Resistance

As an alternative to antibiotics, metal poisoning is used to destroy bacteria (Hao et al., 2016). This has resulted in the emergence and prevalence of AMR, representing a severe threat to public health worldwide (Hammerum and Heuer, 2009). High dietary Cu may have undesired effects, such as the growth of Cu-resistant bacteria (Pang et al., 2009). Copper resistance genes are usually located on plasmids and, in most cases, are transferable (Hasman and Aarestrup, 2002). These plasmids conferring resistance to copper (tcrB) have been identified in several *Enterococcus* species, including *E. faecium* and *E. faecalis*, in pigs, poultry, calves, and also humans (Torres et al., 2018). In *Enterococcus* spp. the most common genes conferring resistance to antibiotics are for erythromycin, tetracycline, and vancomycin (Oravcova et al., 2019; Tian et al., 2019).

On the other hand, there is high diversity and variants of *E. coli* strains integrating the normal gut microbiota and can cause severe diseases in both animals and humans, such as urinary tract infections, diarrhea, enteritis, and septicemia (Li et al., 2019). The routine use of antimicrobials in livestock for either “prophylaxis” or “metaphylaxis” has represented a serious hazard for the selection of multidrug-resistant Enterobacteriaceae strains (Angulo et al., 2004). The effectiveness of treatments against *E. coli* is threatened by the dramatic increase of extended-spectrum beta-lactamases producing isolates worldwide (Livermore et al., 2007; Carattoli et al., 2017).

Regarding Cu resistance genes in the present study, a high prevalence of tcrB was detected in *Enterococcus* spp. isolates mainly on d 42, without any difference between groups. In the case of *E. coli*, tcrB was less frequent, but a higher prevalence was found at d 42. The presence of tcrB is not associated with Cu addition in the diet, as the presence of this gene was higher in the NC group. Another study has reported lower frequencies of tcrB gene in enterococcal isolates (34%) in broiler chickens, but higher levels of prevalence (76%) in pigs (Hasman and Aarestrup, 2002).

The presence of the zinc resistance gene czcA was barely detected in enterococcal isolates in the first two samplings (d 14 and 28). However, in the third sampling (d 42), the prevalence reached 50% in NC. Contrarily, in *E. coli*, the prevalence diminished significantly between samplings, going from 100% at d 14 to 0% at d 42. These differences were observed in all the treatments; therefore, they cannot be correlated with Cu addition in the diet.

Overall, a high prevalence of AMR genes was observed in *Enterococcus* spp. isolates. Erythromycin use is permitted in chickens, and laying hens and tetracyclines have been widely used as a growth promoter in animal husbandry (Granados-Chinchilla and Rodríguez, 2017). Therefore, the high prevalence of ermB and tetM could be explained by the frequent use of these antimicrobials in broiler farms over the years. A high prevalence of ermB and tetM in broiler excreta has been reported before in *Enterococcus* spp. isolates (Cauwerts et al., 2007; Tremblay et al., 2011; Hasan et al., 2018). Also, Cauwerts et al. (2007) found a correlation between the presence of ermB and phenotypical resistance to tetracyclines, mediated by several tet genes, tetM being among them.

The use of avoparcin, whose structure is similar to that of vancomycin, has decreased over the years in animal feed, and it has resulted in a reduction of the number of vancomycin-resistant isolates (Yazdankhah et al., 2014). Regarding the vancomycin resistance genes, high prevalences for the vanC1 and vanC2 genes were detected. The vanC genes are associated with low-level vancomycin resistance (Watanabe et al., 2009; de Moura et al., 2013), and are considered intrinsic in some enterococcal species, such as *E. gallinarum* and *E. casseliflavus* (Monticelli et al., 2018). The presence of these genes could be explained by the presence of this latter species on the excreta samples, and the possible transmission of plasmids containing the resistance genes between enterococcal species. Also, the presence of the vanC gene can explain the intermediate phenotypical resistance found in some of the analyzed isolates.

The phenotypical resistance in Enterococcal and *E. coli* isolates was high in almost all the antimicrobial agents. This high prevalence may be due to the fact that this study was carried in the north-east part of Spain, which has a very high density of pig production, where antimicrobial agents are widely used and the prevalence of AMR genes, principally in *E. coli* strains, has been highly reported (Vidal et al., 2020). Subsequently, observing any significant effect on interventional groups is challenging, given the high background levels of AMR in the non-supplemented group.

Likewise, most of the *E. coli* strains were resistant to vancomycin. This resistance was expected as vancomycin has been designed to kill a different type of microbe: gram-positive cocci. However, there were differences between Cu non-supplemented and supplemented in *E. coli* isolates for streptomycin and chloramphenicol, where birds supplemented with Cu had higher resistance than non-supplemented. Although in Agga et al. (2014) study with pigs, they did not observe any difference in the resistance prevalence of Streptomycin between Cu supplementations or without, but higher chloramphenicol prevalence was found in non-Cu-supplemented pigs. However, the mean prevalence was similar for both studies. For other antimicrobial agents (ampicillin, gentamicin, and kanamycin), the prevalences were similar between both studies, which suggests that Cu supplementation could have a similar effect in antimicrobial resistance for pigs and poultry. Controversially, in the *Enterococcus* isolates, no resistance was observed in vancomycin, and higher resistance prevalence was observed for non-Cu-supplemented birds in enrofloxacin, gentamicin, and chloramphenicol. Therefore, further studies with more controlled environmental conditions should implement in this field to assess the origin of these AMR genes.

## CONCLUSION

To conclude, supplementation of the high dose of dicopper oxide (150 mg Cu/kg) was able to enhance the growth performance of broiler chickens raised under challenging conditions by modulating bacterial communities in the ileum. Finally, Cu addition did not alter the AMR genes in this study, which suggests that using broilers in a reused litter does not seem an appropriate method to check for AMR genes.
